# Construction of High-Density Genetic Map and Identification of a Bruchid Resistance Locus in Mung Bean (*Vigna radiata* L.)

**DOI:** 10.3389/fgene.2022.903267

**Published:** 2022-07-08

**Authors:** Tianxiao Chen, Liangliang Hu, Suhua Wang, Lixia Wang, Xuzhen Cheng, Honglin Chen

**Affiliations:** Institute of Crop Sciences, Chinese Academy of Agricultural Sciences, Beijing, China

**Keywords:** mung bean (*Vigna radiata* L.), SLAF-seq, SNP, genetic map, bruchid resistance

## Abstract

Mung bean (*Vigna radiata* L.) is an economically important grain legume cultivated in Asian countries. High-density genetic linkage is a valuable and effective tool for mapping quantitative trait loci (QTL). In the current study, a high-resolution genetic map containing 4,180 single-nucleotide polymorphisms (SNPs) was assigned to 11 linkage groups (LGs) and spanning 1,751.39 cM in length was constructed for mung bean, and the average distance between adjacent markers was 0.42 cM. Bruchids (*Callosobruchus* spp.) cause significant damage to and loss of legume seeds. A locus for bruchid resistance was detected. The gene *Vradi05g03810*, encoding a probable resistance-specific protein, was found to be the most likely key candidate gene in mung beans. A 69-bp sequence deletion was identified in the coding region by comparing the cDNA sequences of bruchid-resistant and bruchid-susceptible lines. This SNP-based high-density linkage map is one of the first to be constructed across the mung bean genome. This map will not only facilitate the genetic mapping of genes or complex loci that control important agronomic traits but also offer a tool for promoting future genetics and comparative genomic studies in *Vigna*.

## Introduction

Mung bean (*Vigna radiata* [L.] R. Wilczek, 2n = 2x = 22) is an important pulse crop that is cultivated in South and Southeast Asia. It is grown on more than 6 million hectares with a global annual production of 3 million tons (Nair et al., 2013). Mung bean is consumed as dry seeds and as bean sprouts. Seeds are valuable sources of protein and carbohydrate, and sprouts are important sources of vitamins and minerals for human nutrition. Because of its high content of protein, iron, and folate, which provide great nutritional health benefits, mung bean is a nutrient-balanced food for cereal-based diets (Tomooka et al., 2000) and has been a common food product in China for thousands of years (Tang et al., 2014).

However, mung bean production is constrained by destructive insect pests. These insect pests limit the yield potential of grain legumes, inflicting losses of billions of dollars worldwide every year. Among insect pests, bruchids (*Callosobruchus* spp.) are major storage pests that cause serious damage to the seeds of legume grains, including mung bean (*Vigna radiata*), adzuki bean (*Vigna angularis*), cowpea (*Vigna unguiculata*), black gram (*Vigna mungo*), common bean (*Phaseolus vulgaris*), chickpea (*Cicer arietinum*), pigeon pea (*Cajanus cajan*) and others (Southgate, 1979). *Callosobruchus* are the most destructive mung bean pest of the various bruchid species, and these bruchids infest the pods in the field and seeds in storage, which causes weight loss and low germination (War et al., 2017). Host plant resistance is an economical and sustainable way to preserve mung bean seeds against destruction by bruchids. Incorporating host plant resistance against bruchids along with good agronomic traits would be a better way to controlling these pests in mung beans. Therefore, breeding resistant varieties and exploring the resistance mechanism are major goals in mung bean breeding programs. However, only a few cultivated varieties of bruchid-resistant mung beans are available (Somta et al., 2007). To determine the bruchid resistance genotype, thousands of mung bean accessions have been screened for bruchid resistance. To date, several mung bean genotypes with resistance to bruchids are available, including three wild (*V. radiata* var. *sublobata*) and four cultivated (*V. radiata* var. *radiata*) mung bean genotypes. The wild mung bean accession TC 1966 (Fujii and Miyazaki, 1987; Fujii et al., 1989) and cultivated mung bean accessions V1128, V2817, and VC6089A are completely resistant to *C. chinensis* and *C. maculatus*. Two wild mung bean accessions, ACC23 and ACC41, are resistant to both bruchid species (Lambrides and Imrie, 2000), while two cultivated accessions, V2709 and V2802, are highly resistant to *C. chinensis* and *C. maculates* (Talekar and Lin, 1992; Somta et al., 2008). V2709 has been used to breed the bruchid-resistant varieties Zhonglv No. 3, Zhonglv No. 4, and Zhonglv No. 6. These lines containing the resistance gene from V2709 were confirmed to be safe for human consumption based on animal safety and toxicity tests (Yao et al., 2015).

A major locus conferring resistance to bruchids was identified from two highly resistant wild genotypes, TC1966 and ACC41 (Lambrides and Imrie, 2000), and two highly resistant cultivated genotypes, V2709 and V2802 (Somta et al., 2007). Restriction fragment length polymorphism (RFLP) markers have been developed to map resistance genes in both genotypes (Young et al., 1992; Kaga and Ishimoto, 1998; Miyagi et al., 2004; Mei et al., 2009). The bruchid resistance gene (*Br*) in TC1966 is located on LG VIII between the RFLP markers pA882 and pM151a (Young et al., 1992). The *Br* locus in TC1966 has been fine mapped to a 0.7 cM interval between RFLP markers Bng110 and Bng143, and it is only 0.2 cM away from Bng143 (Kaga and Ishimoto, 1998). A few molecular markers for the *Br* locus in ACC41 have been developed, including the RFLP marker mgM213 (1.3 cM) (Miyagi et al., 2004) and the simple sequence repeat (SSR) markers BM202 (0.7 cM) and Vr2-627 (1.7 cM) (Wang et al., 2016). Seven cleaved amplified polymorphism (CAP) markers have been developed and are closely linked with the *Br* locus in TC1966 (Chen et al., 2007). V2709 and V2802 are considered to be a single dominant locus with several modifiers (Somta et al., 2007). The expressed sequence tag-simple sequence repeat (EST-SSR) marker DMB-SSR158 cosegregates perfectly with the *Br* gene in V2802 (Chotechung et al., 2016) and is also tightly linked with the *Br* locus in TC1966 (Chen et al., 2007; Chen H.-M. et al., 2013). Recently, a polygalacturonase inhibitor gene located on chromosome 5 was reported to possibly be responsible for bruchid resistance in V2802 (Chotechung et al., 2016).

SNPs are widely distributed throughout the mung bean genome, and the ability to obtain thousands of SNPs and construct high-density SNP genetic maps has been made feasible by the rapid development of next-generation sequencing (NGS) technology. Several methods, including restriction-site associated DNA tag sequencing (RAD-seq) (Miller et al., 2007), genotyping-by-sequencing (GBS) (Sonah et al., 2013), and specific-locus amplified fragment sequencing (SLAF-seq) (Sun et al., 2013), have been used to discover SNP markers. A high-density linkage map is essential for fine mapping of QTLs/genes and comparative genomics studies. SLAF-seq is a newly developed high-throughput approach for genome-wide SNP detection and genotyping based on NGS.

In the present study, a high-throughput SLAF-seq strategy was employed to construct an SNP-based genetic map for mung bean, and a bruchid resistance-related QTL was identified. Large-scale SNP analysis allowed the detection of a genomic region containing the major bruchid resistance locus, as well as candidate gene identification. Combined with conventional genetic analysis and expression patterns, we provided evidence that *Vradi05g03810*, encoding a probable resistance-specific protein, is a candidate gene for bruchid resistance. The objectives of this study were 1) to finely map bruchid resistance loci, 2) to analyse candidate gene(s) based on the SLAF-seq strategy, and 3) to develop markers closely linked to *Vradi05g03810* conferring bruchid resistance, which will provide SNPs for marker-assisted selection (MAS) in future breeding programs.

## Materials and Methods

### Plant Material and Phenotypic Evaluation

A RIL population consisting of 150 individuals obtained from a cross between the bruchid-susceptible female parent VC2778A and the bruchid-resistant male parent TC 1966, was used for genetic map construction. In the spring of 2014, the seeds of the parents and population were planted in an experimental field of the Chinese Academy of Agricultural Sciences (China, Beijing, 116°46′E, 39°92′N). Young leaves from RIL individuals and the two parents were collected and stored at −80°C until DNA extraction. To confirm the inheritance of bruchid resistance, the mature seeds of VC2778A and TC1966, and those harvested from the RIL population (VC2778A × TC1966) were evaluated against *C. chinensis*. Together with Zhonglv No.1 (susceptible cultivar) as a control, 30 seeds of the F_2_ populations were placed separately into plastic dishes, which were replicated three times for each line. Approximately 500 adult bruchid beetles were released into the box. These boxes were incubated at 28°C and 70% relative humidity. Sixty days later, the number of damaged seeds of each line was counted, and the percentage of damaged seeds was used to classify each line as either resistant or susceptible. The ratio of the number of damaged seeds to the total number of seeds tested was used to calculate the bruchid infestation. Lines that exhibited 0–20% damaged seeds were considered highly resistant (HR), those that exhibited 81–100% damaged seeds were considered highly susceptible (HS), and those that exhibited 21–80% damaged seeds were considered moderately resistant (MR) (Chotechung et al., 2016).

### DNA Extraction and SLAF Sequencing

DNA was isolated and purified using the CTAB (cetyl trimethyl ammonium bromide) method (Attitalla, 2011). The quantity and quality of extracted DNA were assessed using an ND-1000 spectrophotometer (NanoDrop, Wilmington, DE, United States) (Desjardins and Conklin, 2010) and by electrophoresis in 0.8% agarose gels. A subset of 150 F_2_ individuals and the two parents were subjected to SLAF library construction and sequencing. Resistant or highly susceptible pools were constructed by mixing equal amounts of DNA from 10 highly resistant individuals or highly susceptible F_2_ individuals. The DNA isolated from TC1966 and VC2778A and the two DNA pools were used for SLAF library construction and sequencing following a previous report (Sun et al., 2013). We employed a SLAF-seq approach in our experiment. RsaI and HaeIII (New England Biolabs, NEB, United States) were used to digest the genomic DNA. The pooled samples were electrophoresed on a 2% agarose gel, and the fragments between 314 and 394 bp were purified using a QIAquick gel extraction kit (QIAGEN, Hilden, Germany). All the purified products were then diluted and subjected to paired-end sequencing on an Illumina HiSeq 2,500 platform (Illumina, Inc.; San Diego, CA, United States) at Biomarker Technologies Corporation in Beijing.

### Sequence Data Grouping and Genotyping

SLAF-seq data grouping and genotyping were performed as described (Sun et al., 2013)*.* Low-quality reads (quality score <20^*e*^) were filtered out by real-time monitoring, and the high-quality clean reads were clustered based on sequence similarity by BLAT. Sequences with more than 95% identity were grouped into one SLAF marker. The alleles of each SLAF were defined according to those of the parents, and individuals were genotyped based on sequence similarity to their parents. Alleles were defined in each SLAF by minor allele frequency (MAF) evaluation. Mung bean is a diploid species, SLAFs with more than four alleles were discarded, and SLAFs with 2–4 alleles were identified as potentially polymorphic. In this study, SLAFs with a sequence depth of less than 200 were defined as low-depth SLAFs and were filtered out in the subsequent analysis. SLAFs with 2, 3, or 4 tags were identified as polymorphic SLAFs and were considered to be potential markers. Polymorphic SLAFs were analyzed based on the population type, which consisted of eight segregation patterns (aa × bb, ab × cd, ef × eg, ab × cc, cc × ab, hk × hk, lm × ll, and nn × np). In our study, an F2 population was used; therefore, the segregation pattern aa × bb was selected for subsequent analysis. A Bayesian approach was used to evaluate the genotyping quality for genotype scoring (Sun et al., 2013).

### Genetic Map Construction

The high-density linkage map was constructed using HighMap software (Liu et al., 2014). The HighMap strategy was utilized to correct the incorrect SNP marker orders within each LG caused by genotyping errors. MLOD scores >5 between markers confirmed the robustness of markers for each LG. SMOOTH algorithms were used to correct genotyping errors. Using the Kosambi mapping function, recombination percentages were converted to genetic distance. Finally, the map quality was evaluated by heatmaps and haplotype maps.

### Comparative Genome Analysis

The 341,502 SLAF markers generated from mung beans were aligned to the genome sequences of the common bean (Phaseolus vulgaris) and adzuki bean (Vigna angularis) using BLASTN (version 2.2.30) (Altschul et al., 1990), with an e-value cut-off of ≤1e^−5^. Their GenBank assembly accessions were GCA_001517995.1 and GCA_001723775.1, respectively. SLAF markers with query sequence identities >98% and sequence lengths >80 were selected. Syntenic gene blocks within the genome were detected by MCScanX (Wang et al., 2012) and visualized using the jcvi python module.

### Association Analysis and Candidate Gene Selection

Equal amounts of genomic DNA from 15 highly resistant and 15 highly susceptible RILs were separately pooled to make resistant and susceptible bulks. The sequence data of the two parents and resistant and susceptible bulks were generated by Illumina HiSeq 2500. A SNP index was calculated. Differences in the genotype frequencies of the pooled samples were determined by the SNP index (Abe et al., 2012), and Δ(SNP-index) values were calculated by subtracting the bruchid susceptibility index from the bruchid resistance index at each SNP position (Takagi et al., 2013).

The genes in the causal region were detected according to the functional annotation of the reference genome of mung bean. The amino acid sequence polymorphisms between VC2778A and TC1966 were screened and aligned back to the reference genome. Only those genes with at least one amino acid difference were considered candidates. The candidates were then compared with the NR to obtain a more detailed functional annotation database.

### Sequence Variance Analysis by an InDel Marker

An InDel marker was identified by comparison of resistance-specific protein (*RSP*) sequences between the bruchid-resistant and bruchid-susceptible lines, and degenerate primers for amplification were designed by Primer Premier 5.0 based on the sequence differences between TC1966 and VC2778A. The partial sequence of *RSP1* was amplified using primers: 5′- TCA​AAC​AGC​CAA​GCT​AAA-3′ and 5′-CGCCTCAATCYTTCTTCTG-3’. PCR reactions were conducted using the following program: 5 min at 95 °C, 30 s at 95 °C, 30 s at 55 °C, and 40 s at 72 °C.

## Results

### SLAF Sequencing and SNP Markers

A total of 36.18 GB of high-quality sequence data containing 183.34 M 100-bp paired-end reads were generated after SLAF library construction and sequencing. The average percentage of Q30 bases (a quality score of 30) was 85.94% for all reads, and the average guanine-cytosine (GC) content was 36.96%. The numbers of SLAF markers in the male parent and female parent were 235,157 and 267,294, respectively, with an average depth (the average number of reads in each SLAF) of 27.19-fold and 27.41-fold, and the average depths of each SLAF marker was 26.91-fold and 27.50-fold. For the F_2_ population progeny, the number of SLAFs ranged from 126,056 to 174,509, with an average value of 148,091 and a mean depth of 5.74-fold, and the individual integrity of the mapped markers (the ratio of known genotypes to total markers) was 95.06%. Overall, a total of 341,502 high-quality SLAF markers were detected, and 179,690 were polymorphic, resulting in a polymorphism rate of 52.62% (Table 1). The 179,690 polymorphic SLAFs harboured a total of 775,853 SNPs. A total of 541,768 SNPs were generated in the male parent, and the average integrity of each SNP marker was 69.82%. A total of 574,722 SNPs were generated in the female parent, with an average integrity of 74.07% for each SNP. In the RIL mapping population, a total of 305,236 SNPs were generated, and the average integrity was 39.34% for each individual. These high-quality SNPs were classified into eight segregation patterns. Since the two parents (TC1966 and VC2778A) used for genetic map construction in the F_2_ population were homozygous mung bean lines with genotypes of aa and bb, the multiallelic markers with tag numbers larger than 4 were filtered out; 131,340 (16.93%) SNPs fell into this class.

**TABLE 1:** High-quality data generated by sequencing the SLAF library.

Samples	No. of Clean Reads	GC Content (%)	Q30 (%)	No. of SNPs	No. of SLAFs	Total Depth	Average Depth
TC1966	8,581,649	36.30	85.76	541,768	267,294	7,350,585	27.50
VC2778A	9,128,859	36.97	85.68	574,722	235,157	6,328,075	26.91
Progenies	1,104,205	36.97	85.95	305,236	148,091	845,110	5.74

### Characteristics of the Genetic Maps

**FIGURE 1:**
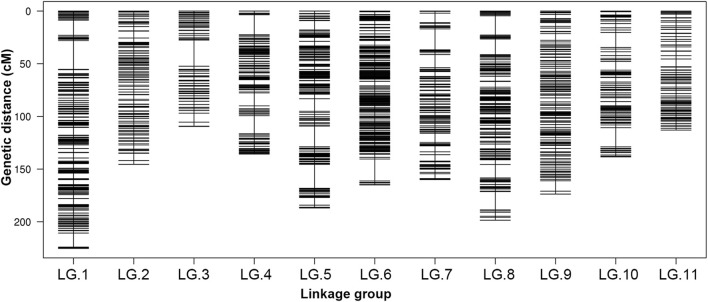
Distribution of SLAF markers on 11 LGs of mung bean. A black bar indicates a marker. The *x*-axis represents linkage group number and the *y*-axis indicates genetic distance (cM).

After a five-step filtering process, 4,249 polymorphic SNP markers were used for high-density genetic map construction using HighMap software (Figure 1). A total of 4,180 (98.38%) of the 4,249 SNPs were mapped to the genetic map, and the final map spanned a total of 1,751.39 cM in length with a mean intermarker distance of 0.42 cM. The number of markers mapped to each LG ranged from 113 in LG.3 to 748 in LG.6, with an average of 380 markers per LG. The length of the individual LGs varied from 109.55 to 225.26 cM, with average distances of 0.22–1.06 cM between adjacent markers (Table 2). The largest linkage group, LG.1, harboured 598 markers spanning a length of 225.26 cM, and the average intermarker distance was 0.38 cM. The smallest linkage group, LG.3, contained 113 markers spanning 109.55 cM with an average intermarker distance of 0.98 cM. LG.6 was the densest, whereas LG.11 contained the fewest markers.

**TABLE 2:** Summary of the high-density genetic map constructed in mung bean.

Linkage group	Total Number of Markers	Length (cM)	Average Distance Between Adjacent Markers (cM)	Gap<5 cM^a^ (%)	Largest Gap (cM)
LG.1	598	225.26	0.38	98.16%	27.28
LG.2	176	145.50	0.83	98.86%	6.81
LG.3	113	109.55	0.98	97.32%	24.79
LG.4	263	135.74	0.52	97.71%	18.87
LG.5	619	186.85	0.30	98.38%	22.63
LG.6	748	164.97	0.22	99.87%	20.84
LG.7	359	160.00	0.45	94.13%	19.83
LG.8	559	198.44	0.36	98.39%	18.21
LG.9	412	173.70	0.42	99.03%	9.92
LG.10	225	138.40	0.62	98.21%	18.15
LG.11	108	112.98	1.06	99.07%	7.29

a Gap < 5 cM: the percentages of gaps in which the distance between adjacent markers is smaller than 5 cM.

### Comparative Genome Analysis

The 4,249 SNP markers generated from mung bean were used for comparative analysis with the genome sequences of mung bean (*Vigna radiata*), adzuki bean (*Vigna angularis*), and common bean (*Phaseolus vulgaris*). The total numbers of matched markers containing sequences in mung bean and each of the other species were 4,180 (98.38%) for mung bean, 3,050 (71.78%) for adzuki bean, and 907 (4.30%) for common bean. These results indicated that mung bean is more closely related to adzuki bean than common bean. A Circos plot was generated to show the relationship between mung bean, common bean, and adzuki bean (Supplementary Figure S1), which indicated that the SNP genetic map constructed had sufficient coverage across the mung bean, adzuki bean, and common bean genomes, demonstrating a correspondence between the mapped SNP markers and their genomic locations. Most of the SNPs on the linkage map were in the same order as those on the corresponding physical map of the mung bean genome. The adzuki bean genome showed better compatibility with the physical map of the mung bean than with that of the common bean.

### Phenotypic Variation and Association Analysis

**FIGURE 2:**
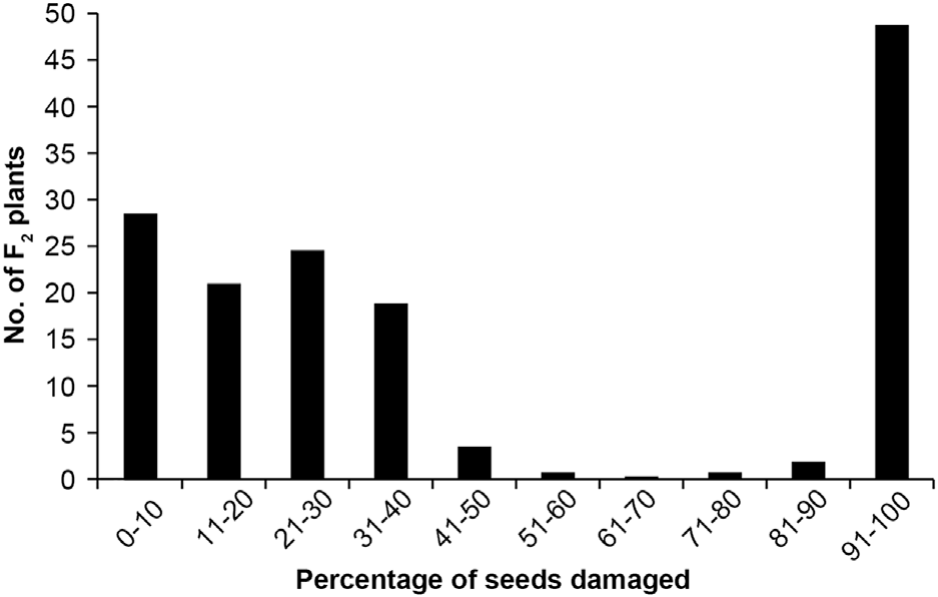
Frequency distribution of the percentage of seeds damaged.

As expected, the seeds of TC1966 and VC2778A differed greatly in their resistance to bruchids. The seeds of TC1966 appeared symptom-free, whereas the susceptible parent, VC2778A, showed worm-eaten symptoms. The percentage of damaged seeds was noncontinuous in the F_2_ population, ranged from 0 to 100% with a mean of 45.31% (Figure 2).

**FIGURE 3:**
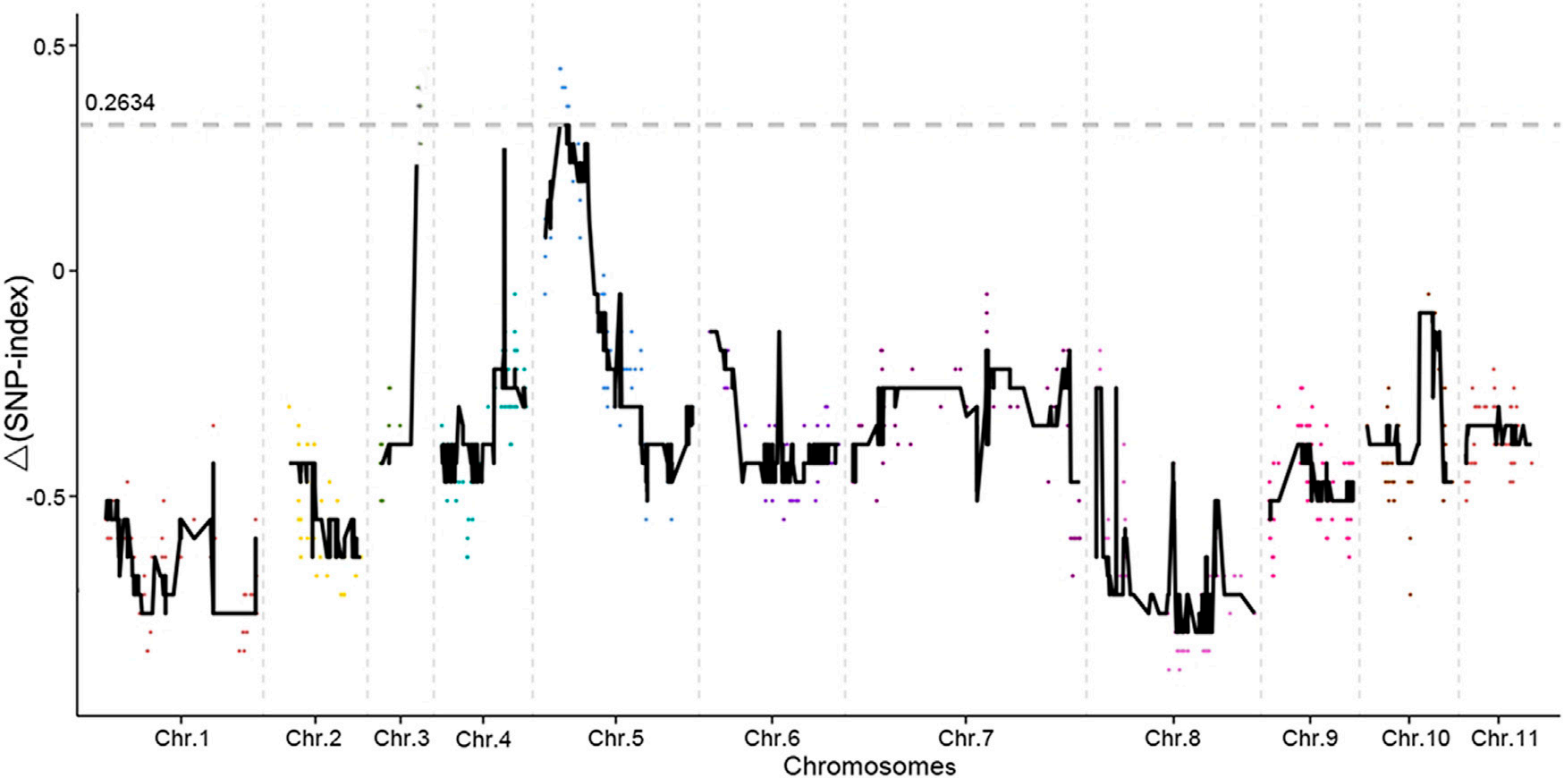
Results of genetic association analysis to identify regions of the mung bean genome associated with resistance to bruchid infestation. The *x*-axis shows the physical positions of the SLAF marker in different chromosomes. The *y*-axis shows the Δ (SNP-index) of the SLAF marker. Every color dot represents Δ(SNP-index) of the SLAF marker in every chromosome. The black lines are the loess fitting curve of Δ(SNP-index) based on smooth values. The grey dotted line is the threshold of the smooth value of Δ (SNP-index).

SNP-index methods were used for the association analysis in this study. In the Δ(SNP-index) plot, peak regions above the threshold value were defined as those for which the Loess-fitted values were greater than the standard deviations above the genome-wide median. Two candidate regions associated with mung bean bruchid resistance were identified, and the average Δ(SNP-index) value above the threshold was 0.2634 (Figure 3). One candidate region associated with mung bean bruchid resistance spanned 0.25 Mb (5.23–5.48 Mb on chromosome 5 of the mung bean VC 1973A reference genome assembly).

### Identification of Candidate Genes for Bruchid Resistance

**FIGURE 4:**
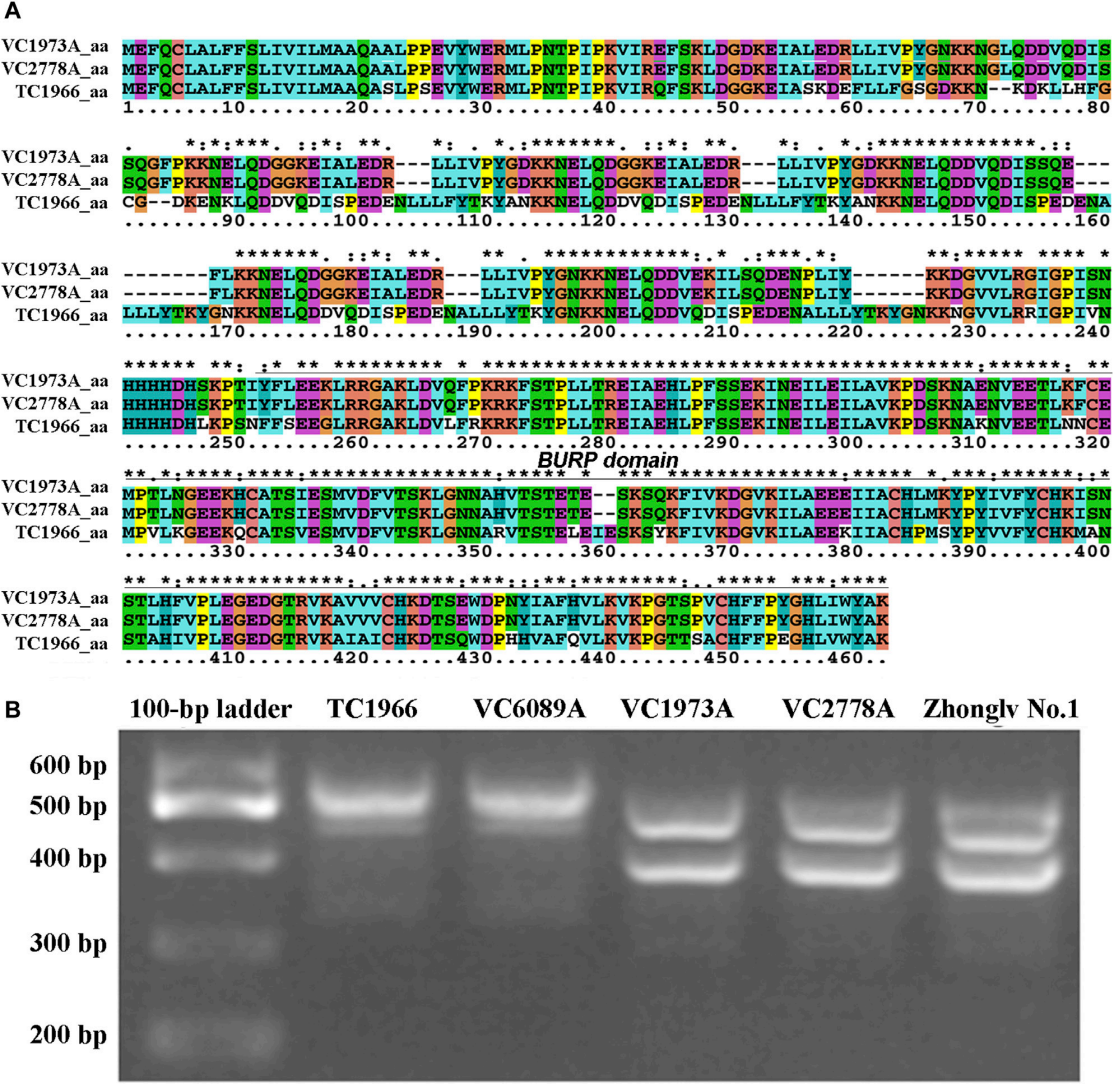
Sequence analysis of *RSP1* gene. **(A)** Comparison of the amino acid sequences of bruchid-resistant and susceptible lines. **(B)** PCR detection of InDel marker in bruchid-resistant lines (TC1966 and VC6089A) and susceptible lines (VC 1973A, VC2778A, and Zhonglv No.1).

A total of 15 genes that encode predicted proteins were detected based on their annotation in the mung bean genome (https://legumeinfo.org/gbrowse_vigra1.0) (Table 3). These genes included seven resistance-specific proteins (RSPs) and two receptor-like protein kinases, while six genes could not be annotated using the known annotation databases. To verify whether these genes were candidate genes, the CDS and genomic DNA sequences were cloned and sequenced from the two parents. CDS comparisons of resistant-specific proteins (*Vradi05g03810*) showed that the sequences were 1,377 bp in the bruchid-resistant lines TC1966 and VC6089A and 1,308 bp in the bruchid-susceptible lines VC2778A, VC 1973A and Zhonglv No. 1 (Supplementary Figure S2). Moreover, the genomic DNA sequence comparisons of *Vradi05g03810* showed that the sequences were 1,806 bp in the bruchid-resistant lines TC1966 and VC6089A and 1,749 bp in the bruchid-susceptible lines VC2778A, VC 1973A and Zhonglv No. 1 (Supplementary Figure S3). The amino acid sequences were significantly different between the two parent lines (Figure 4A). As expected, the InDel marker amplified fragments of 529 bp and 478 bp in TC1966 and VC2778A, respectively, and showed clear polymorphism between the parents (Figure 4B).

**TABLE 3:** Annotation of candidate genes for bruchid resistance identified on Chromosome 5 in mung bean.

Location	Gene ID	Annotation
5,236,102–5,238,685	Vradi05g03810	Resistant specific protein-1 (4)
5,236,102–5,238,685	Vradi05g03820	Hypothetical protein
5,284,760–5,286,543	Vradi05g03830	Resistant specific protein-2
5,300,271–5,304,169	Vradi05g03840	Resistant specific protein-2
5,338,888–5,339,265	Vradi05g03850	Hypothetical protein
5,367,245–5,367,592	Vradi05g03860	Resistant specific protein-2
5,389,200–5,390,826	Vradi05g03870	Resistant specific protein-1 (4)
5,390,006–5,391,091	Vradi05g03880	Resistant specific protein-1 (4)
5,409,399–5,413,153	Vradi05g03890	Hypothetical protein
5,452,580–5,453,434	Vradi05g03900	Hypothetical protein
5,477,715–5,478,776	Vradi05g03910	Uncharacterized protein
5,484,862–5,485,213	Vradi05g03920	Uncharacterized protein
5,511,812–5,514,336	Vradi05g03930	Resistant specific protein-2
5,560,120–5,563,268	Vradi05g03940	Polygalacturonase inhibitor
5,590,573–5,598,731	Vradi05g03950	Polygalacturonase inhibitor

## Discussion

High-density linkage maps are essential tools for gene mapping, the identification of candidate genes, and the comparative analysis of synteny (Wang et al., 2011; Xu et al., 2015). However, most genetic maps for mung bean contain only several hundred markers, largely due to genotyping costs and marker discovery technologies (Liu et al., 2014), and the relatively low marker density makes mapping QTLs/genes difficult. Some genetic linkage maps of mung bean have been constructed using several conventional molecular markers, such as RFLPs (Humphry et al., 2002), RAPDs (Lambrides et al., 2000), ISSRs (Khajudparn et al., 2012), and SSRs (Wang et al., 2016). These markers have been used to map genes for mung bean weevil resistance (Young et al., 1992; Kaga and Ishimoto, 1998; Mei et al., 2009) and seed color (Lambrides et al., 2004) and to identify QTLs for seed weight (Humphry et al., 2005; Mei et al., 2009), yellow mosaic India virus (MYMIV) resistance (Kitsanachandee et al., 2013), chasmogamous flower (Chen et al., 2016), hard-seededness (Humphry et al., 2005), powdery mildew resistance (Young et al., 1993; Chaitieng et al., 2002; Humphry et al., 2003; Li et al., 2014), domestication-related traits (Isemura et al., 2012), seed starch content (Masari et al., 2017), phytic acid and phosphorus contents (Sompong et al., 2012), and *Cercospora* leaf spot resistance (Chankaew et al., 2011).

Many genetic linkage maps have been developed in mung bean. Due to the use of fewer molecular markers in mung beans, there are large distances between adjacent markers in some linkage groups, and some reported linkage groups do not coincide with the number of chromosomes in mung beans. We previously mapped loci for bruchid resistance using a genetic linkage map based on 560 marker sets and 210 RILs derived from a cross between Berken × ACC41 (Wang et al., 2016). However, the low polymorphism rate of available molecular markers and their limited quantity made constructing a high-density genetic linkage map more difficult. In addition, the obtained RFLP and RAPD markers were not sufficient because they were either not polymorphic or not easily obtained. SNPs are highly abundant and distributed throughout the whole genome in various plants. Therefore, the construction of a high-density genetic linkage map in mung beans is preferable.

In this study, an SNP-based high-density genetic linkage map was constructed in mung bean using SLAF sequencing technology. We previously constructed a saturated SSR map in mung bean with 560 SSRs and a mean marker interval of 1.3 cM (Wang et al., 2016). The SNP map developed herein contains 4,180 SNPs, and most were anchored to the VC1973A draft genome. Compared with the previous SSR genetic map, the number of markers (560 SSRs vs. 4,180 SNPs), the marker density (1.3 vs. 0.42 cM), and the total map length (732.9 cM vs. 1,751.39 cM) were improved in the SNP genetic map. Compared with a previously constructed genetic map (Isemura et al., 2012), the number of mapped loci (430 vs. 4,180), the marker density (1.78 vs. 0.42 cM), the total map length (727.6 cM vs. 1,751.39 cM) and the average distance between adjacent markers (1.42 cM (LG.1) to 2.11 cM (LG.9) vs. 0.22 cM (LG.6) to 1.06 cM (LG.11) were improved in our new genetic linkage map. Moreover, the number of markers used in this map was substantially higher than that used in the SNP maps previously constructed to assemble the mung bean draft genome (Kang et al., 2014).

High-density polymorphic SNP markers are currently used in genome-wide genotyping and high-density genetic map construction. Advances in NGS technology have made the detection of large numbers of SNP markers across the whole genome possible, as well as the high-throughput identification and genotyping of SNPs at a relatively low cost. NGS-based genotyping methods, such as RAD-seq (Wu et al., 2014), GBS-seq (Spindel et al., 2013), and SLAF-seq (Sun et al., 2013), allow millions of genome-wide SNPs to be identified across the entire plant genome. To date, whole-genome sequencing of mung bean has been completed, and the most saturated mung bean genetic map, which includes 1,321 SNPs, was constructed through GBS-seq (Kang et al., 2014). Compared with these traditional methods for developing markers, SLAF sequencing is a rapid, accurate, and cost-effective method for developing molecular markers (Sun et al., 2013). The SLAF sequencing method has been successfully used in many plants, including soybean (Li et al., 2014), cucumber (Xu et al., 2015), sesame (Zhang et al., 2013), and orchardgrass (Zhao et al., 2016), for molecular marker development and genetic map construction. SLAF-seq genotyping results can also provide important information for molecular-assisted breeding and genome evolution studies and SLAF-seq is an effective method for genome-wide SNP discovery, genotyping, and the fine mapping of QTLs. This method has recently been used successfully to draft genomes (Huang et al., 2013), study genome evolution (Chen S. et al., 2013), and construct high-density genetic maps in various plant species (Xu et al., 2014; Ma et al., 2015; Zhang et al., 2015; Zhao et al., 2016). The high accuracy and low cost of SLAF-seq make this method accessible for molecular plant breeding. The application of this method to gene mapping in mung bean has not been reported. Therefore, this study is the first attempt to construct a high-density SNP genetic map and identify genes for bruchid resistance in mung bean. In total, 341,502 high-quality SLAF markers were developed, and 179,690 of these were polymorphic. A genetic map containing 4,180 SNP markers was developed in this study, and most of these markers were anchored to the mung bean draft genome. The map is composed of 11 LGs, which corresponds to the chromosome number for mung bean*.* SLAF-seq is an ideal technology for developing chromosome-specific molecular markers in mung beans due to its high marker quality and quantity when creating a genetic map.

The bruchid is the most devastating storage pest of grain legumes and causes considerable losses to the mung bean industry. Understanding the molecular mechanisms underlying bruchid resistance is the most effective way to reduce grain legume loss and improve the quality of grain legumes. Mapping bruchid resistance-related genes and developing linked markers will facilitate the marker-assisted selection in mung bean breeding. Thus, this map will be important for cultivar improvements. Great efforts have been made to map *Br* genes in mung beans. Bruchid resistance is controlled by a single dominant locus that was first proposed in TC1966; subsequently additional minor modifier factors were postulated (Sarkar et al., 2011; Chen H.-M. et al., 2013). Bruchid resistance in TC1966 has been mapped previously. The *Br* gene in TC1966 was mapped between two RFLP markers, Bng143 (Vr05: 5,049,905) and Bng110 (Vr05: 6,780,603), on chromosome 5 of the mung bean reference genome (Kaga and Ishimoto, 1998). Later, the *Br* gene was mapped between 6,743,539 and 6,745,030 on chromosome 5 and was associated with the RAPD marker fragment OPW02a4 (Chen et al., 2007). Others have located a similar QTL approximately 16 cM away on chromosome 5 (Schafleitner et al., 2016). One major and two minor QTLs in TC1966 have been reported. An SSR marker, DMB-SSR158, is closely linked to the major QTL on chromosome 5, which is located either in or very near the QTL interval on chromosome 5 (Schafleitner et al., 2016). In our study, we identified one major QTL for bruchid resistance, and this QTL was localized within 5.23–5.59 Mb on chromosome 5, which is in agreement with previous reports (Chen et al., 2007).

The nature of the *Br* gene has been studied. It has been reported that the *Br* gene may be associated with vignatic acid biosynthesis (Kaga and Ishimoto, 1998), 7S storage globulins (vicilins) (Khan et al., 2003), the cysteine-rich protein VrD1 (Chen et al., 2002), chitinase, beta-1,3-glucanase and peroxidase (Khan et al., 2003). The polygalacturonase inhibitor gene has been suggested to be responsible for bruchid resistance in mung bean (Chotechung et al., 2016). In our study, *RSP1* was suggested to be responsible for bruchid resistance, is different from a previous report suggesting that a polygalacturonase inhibitor (*PGIP*) gene may be responsible for bruchid resistance in mung bean (Chotechung et al., 2016). The *RSP1* gene contains conserved motifs for the BURP domain protein. Amino acid sequence alignment showed that there was a significant difference between bruchid-resistance and bruchid-susceptible lines, suggesting that the function of RSP alleles in bruchid-resistance and bruchid-susceptible lines may be different. There are two amino acid deletions and some amino acid substitutions in the BURP domain, and these amino acids may be responsible for bruchid resistance. Further studies that focus on molecular characterization may provide genetic resources to improve the bruchid resistance of mung beans.

## Conclusion

In conclusion, we used a cost-effective, high-throughput, and superior quality SLAF-seq strategy to generate an SNP-based genetic map for mung bean. This map contained 4,180 high-quality SNPs and spanned 1,751.39 cM with a mean marker interval of 0.42 cM. The combination of SLAF-seq and SNP index analysis provides an efficient method for identifying genomic regions related to bruchid resistance. An ∼0.36 Mb hotspot region with 15 candidate genes on Chr. 5 was shown to be tightly associated with bruchid resistance. These candidate genes will be validated for their functions via transformation and mutation approaches in the future studies.

## Data Availability

The original contributions presented in the study are included in the article/Supplementary Material; further inquiries can be directed to the corresponding authors.
