# Supplementary figures and images for: Construction of High-Density Genetic Map and Identification of a Bruchid Resistance Locus in Mung Bean (Vigna radiata L.)
Source: Front Genet. 2022 Jul 8;13:903267. doi: 10.3389/fgene.2022.903267 (PMC9305327; doi:10.3389/fgene.2022.903267)

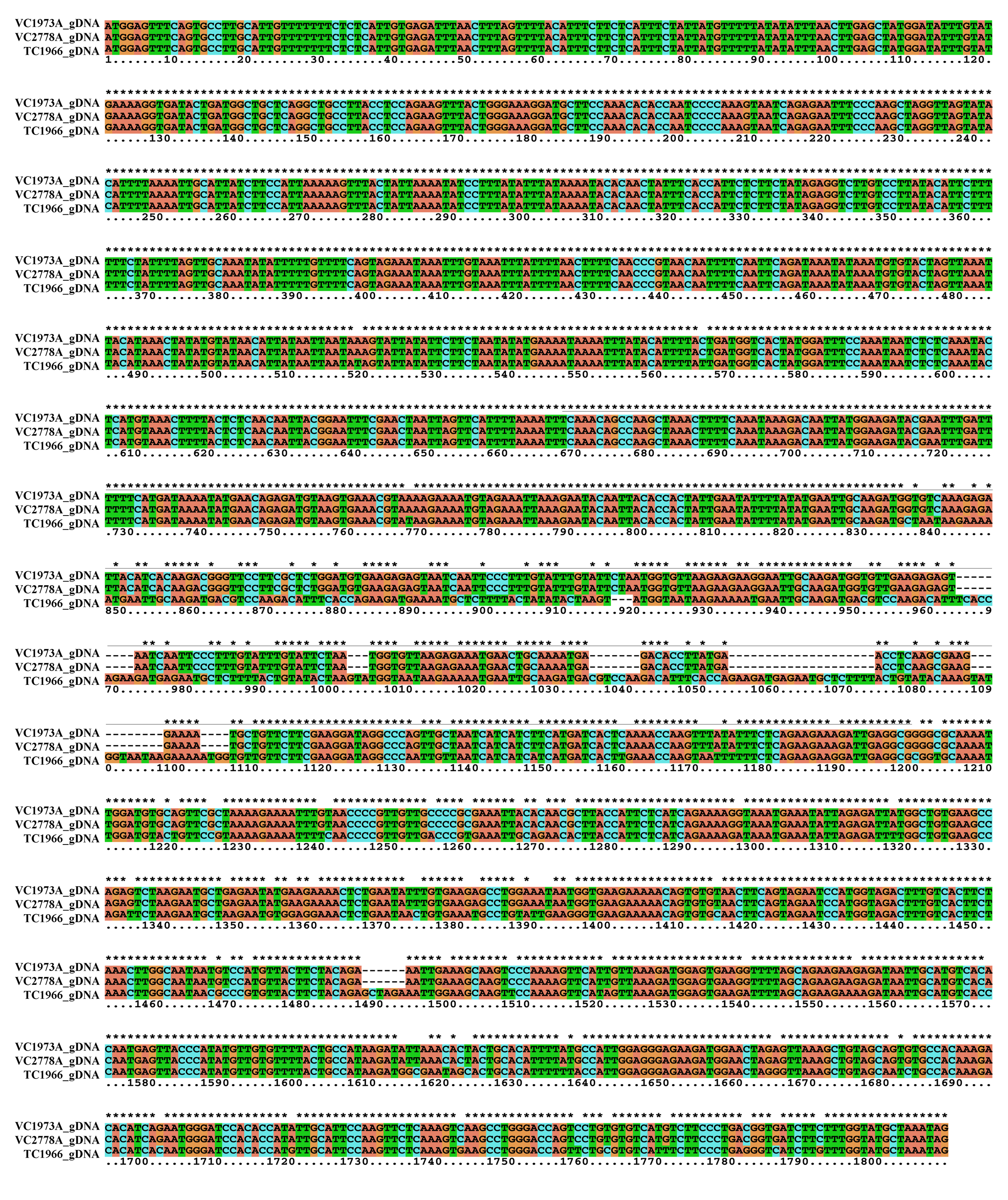

Supplement: Supplementary file 1 [file Image3.tif]

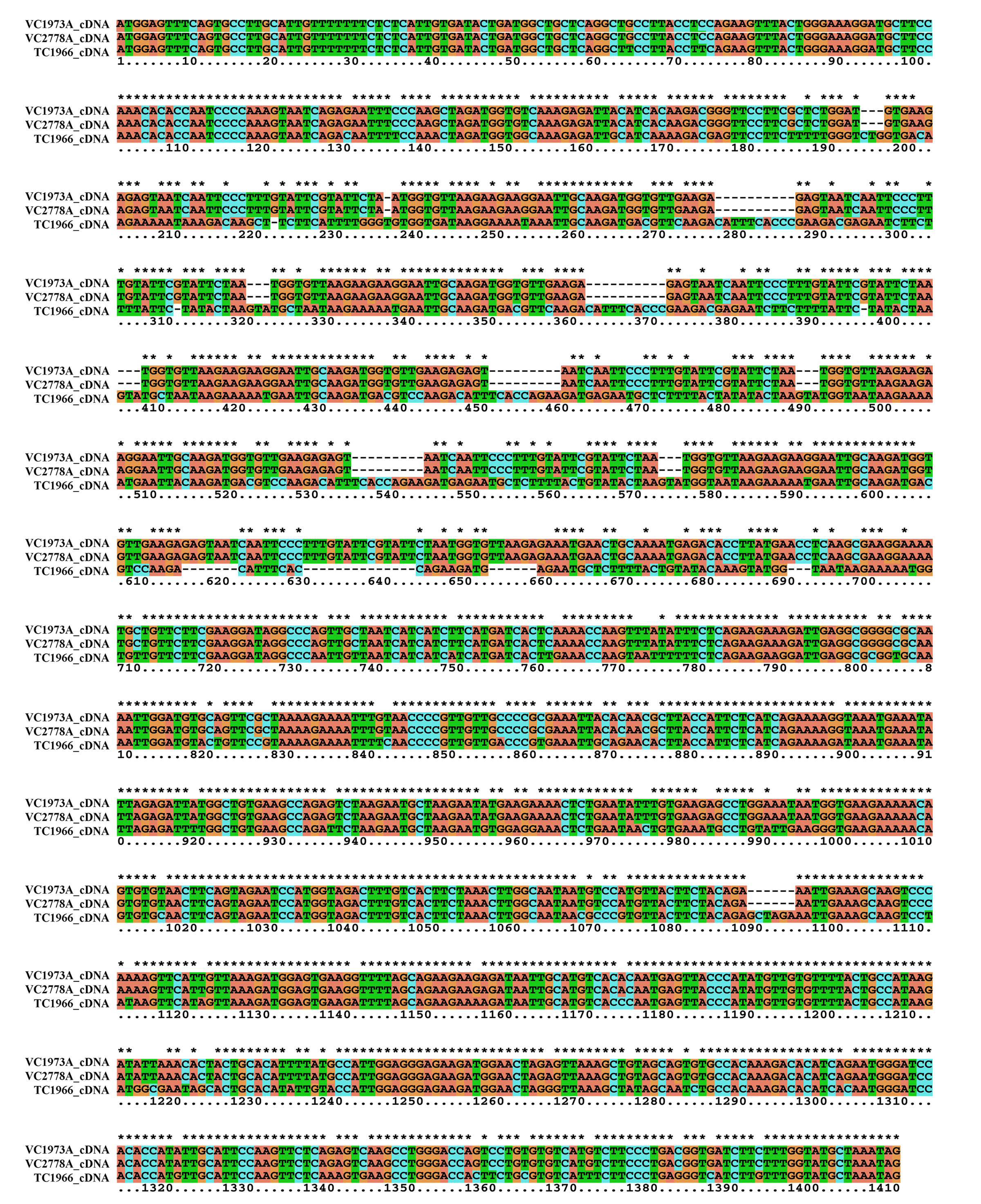

Supplement: Supplementary file 2 [file Image2.tif]

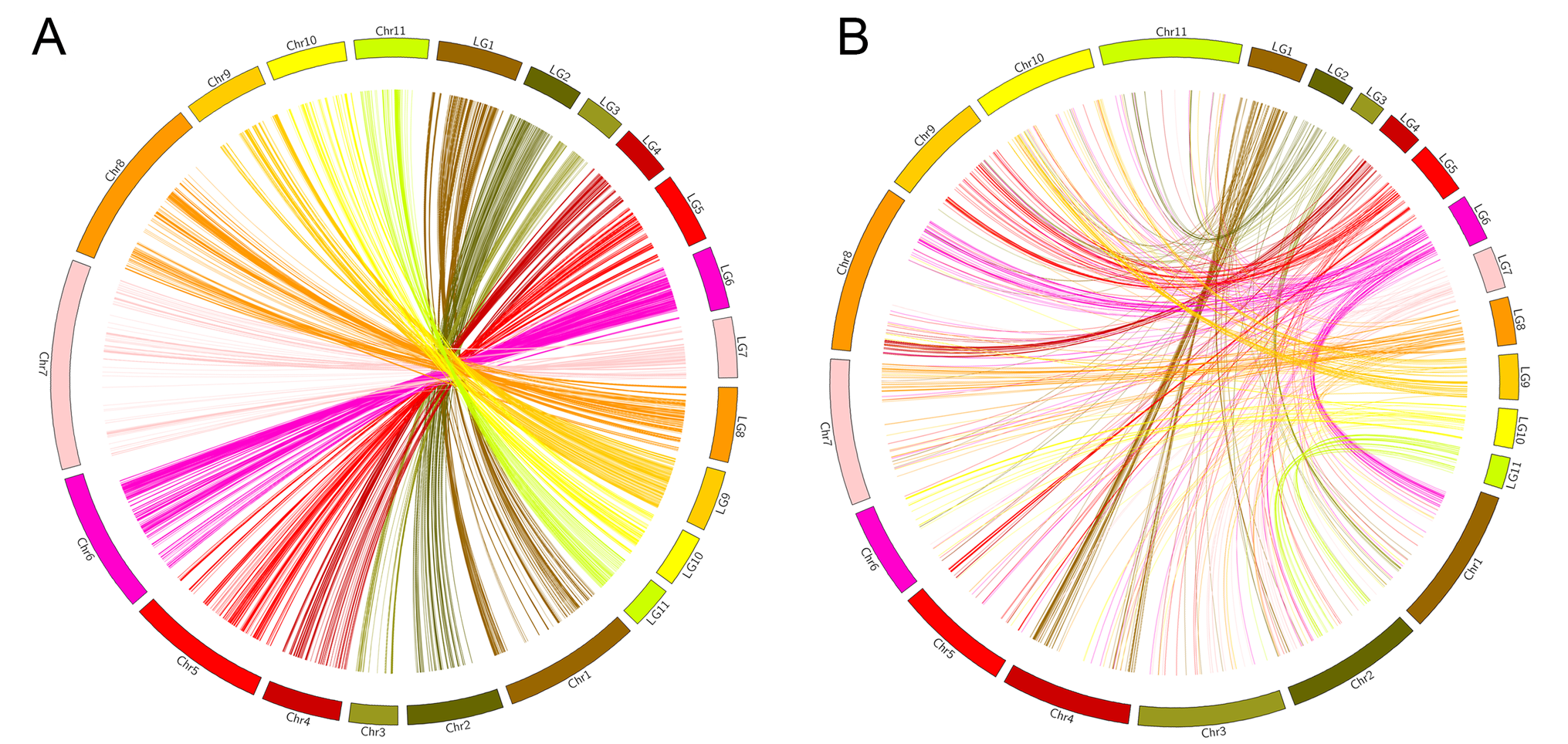

Supplement: Supplementary file 3 [file Image1.tif]
